# Can tomato juice be used for prophylaxis in recurrent stone formers?

**Published:** 2009

**Authors:** Madhu S. Agrawal, Sanjeet Kumar Singh

**Affiliations:** Urology Division, SN Medical College, Agra, India E-mail: dr.madhu.agra@gmail.com

## SUMMARY

In this study the authors have prospectively analyzed the results of 40 samples from tomato, orange, lemon, and mandarin juices. Ten samples of 100 ml were collected from four juice groups. Citrate, oxalate, calcium, phosphorus, magnesium, sodium, potassium, chloride, and pH levels were examined in all samples. The same values were also examined after the samples were stored at 4°C for 1 week.

In fresh juice groups, in tomato juice statistically higher level of citrate (*P* < 0.001, *P* < 0.001, and *P* < 0.001, respectively), higher level of magnesium (*P* < 0.006, *P* < 0.009, *P* < 0.009), and lower level of sodium (*P* < 0.008, *P* < 0.009, *P* < 0.008) were found as compared to orange, lemon, and mandarin juices. No differences were found with regard to calcium, potassium, phosphorus, chloride, and pH in these juices.

Similarly, in stored juice groups, in tomato juice statistical higher level of citrate (*P* <0.001, *P* < 0.001, and *P* < 0.001, respectively), higher level of magnesium (*P* < 0.007, *P* < 0.009, *P* < 0.008), and lower level of sodium (*P* < 0.008, *P* < 0.011, *P* < 0.008) were found as compared to orange, lemon, and mandarin juices. No differences were found with regard to calcium, potassium, phosphorus, chloride, and pH in these juices.

In fresh juice group, statistically lower level of oxalate (*P* < 0.007, *P* < 0.008, *P* <0.006), were found in tomato juice as compared to orange, lemon, and mandarin juices. Higher level of oxalate was found in stored group as compared to fresh juice group (*P* <0.005).

## COMMENTS

Citrate is one of the best known inhibitors of calcium-based stones. Citrate, with its strong anionic nature, binds calcium and forms a soluble salt. As a result, free ionic calcium concentration decreases.[1] Hess *et al*, demonstrated that citrate restored the inhibitory reactivity of Tamm-Horsfall protein in stone formers.[2] Increase in the urine pH also increases ionization of uric acid into more soluble urate anions.[2,3] Citrate also prevents stone formation in other ways, like its ability to adhere on calcium oxalate and phosphate crystals to prevent agglomeration, nucleation, and crystal development; increases urine pH when oxidized into bicarbonate, slows renal citrate metabolism to interfere with citrate reabsorption and causes additional citraturic response.[4]

In recurrent stone formers, potassium citrate is prescribed in doses of 1 meq/kg body weight, approximately 60 meq per day, as long-term prophylactic therapy. However, in medical management for stone prophylaxis patient compliance is generally low as potassium citrate has ulcerogenic potential and may cause gastrointestinal symptoms such as nausea, vomiting, diarrhea, and epigastric pain. These pharmacologic additions also bring about a serious financial burden.[3] The use of potassium citrate together with a potassium-holding diuretic such as triamterene, spironolactone, or amiloride may cause severe hypercalemia.[5]

The high citric acid content of citrus fruits is known to have the potential to increase urine citrate excretion. Citrate that is consumed with the diet is absorbed immediately and almost completely (96 to 98%) through the gastrointestinal tract. More than 90% of the citrate that is absorbed is metabolized. Approximately 10% is excreted in urine without being metabolized.[1]

In this study, authors have shown higher level of citrate in tomato as compared to citrus plants, which are natural sources of citrate. In addition, the lower level of oxalate as compared with that in citrus fruits is a noteworthy advantage. The high level of magnesium and low level of sodium and oxalate, particularly in fresh tomato juice, is also remarkable. In terms of calcium and pH levels, no significant differences were observed between tomato and citrus fruits. The amount of oxalate was shown to increase in stored tomato juice.

All above factors, that is higher citrate and magnesium levels as well as lower sodium and oxalate levels help in prevention of stone formation. Fresh tomato juice is well tolerated and inexpensive. The results of this study indicate that, following further human studies, fresh tomato juice may be recommended instead of pharmacologic potassium citrate for prophylactic purposes in mild to moderate hypocitraturic recurrent nephrolithiasis.

Interestingly, in India, there is widespread misconception among general population as well as among general practitioners that tomatoes are high in oxalate, and predispose to stone formation. This study convincingly demonstrates the truth to be just the opposite, and tomato juice may prove to be the treatment of choice in stone prevention.
